# A Phase I Study of Unimolecular Pentavalent (Globo-H-GM2-sTn-TF-Tn) Immunization of Patients with Epithelial Ovarian, Fallopian Tube, or Peritoneal Cancer in First Remission

**DOI:** 10.3390/cancers8040046

**Published:** 2016-04-22

**Authors:** Roisin E. O’Cearbhaill, Govind Ragupathi, Jianglong Zhu, Qian Wan, Svetlana Mironov, Guangbin Yang, Maria K. Spassova, Alexia Iasonos, Sara Kravetz, Ouathek Ouerfelli, David R. Spriggs, Samuel J. Danishefsky, Paul J. Sabbatini

**Affiliations:** 1 Gynecologic Medical Oncology Service, Department of Medicine, Memorial Sloan Kettering Cancer Center, New York, NY 10065, USA; KravetzS@mskcc.org (S.K.); spriggsd@mskcc.org (D.R.S.); sabbatip@mskcc.org (P.J.S.); 2 Department of Medicine, Weill Cornell Medical College, New York, NY 10065, USA; 3 Melanoma & Immunotherapeutics Service, Department of Medicine, Memorial Sloan Kettering Cancer Center, New York, NY 10065, USA; ragupatg@mskcc.org; 4 Chemical Biology Program, Memorial Sloan Kettering Cancer Center, New York, NY 10065, USA; Jianglong.Zhu@utoledo.edu (J.Z.); wanqian@hust.edu.cn (Q.W.); yangg4@mskcc.org (G.Y.); mspassova1@gmail.com (M.K.S.); o-ouerfelli@ski.mskcc.org (O.O.); s-danishefsky@ski.mskcc.org (S.J.D.); 5 Department of Radiology, Memorial Sloan Kettering Cancer Center, New York, NY 10065, USA; radlana22@gmail.com; 6 Department of Epidemiology and Biostatistics, Memorial Sloan Kettering Cancer Center, New York, NY 10065, USA; iasonosa@mskcc.org

**Keywords:** peptide vaccine, unimolecular vaccine, QS-21, ovarian cancer, remission, immunogenicity

## Abstract

We conducted a phase I study in ovarian cancer patients to evaluate the safety and immunogenicity of a synthetic unimolecular pentavalent carbohydrate vaccine (Globo-H, GM2, sTn, TF, and Tn) supported on a peptide backbone, conjugated to keyhole limpet haemocyanin (KLH), and mixed with immunological adjuvant QS-21. Twenty-four advanced-stage, poor-risk, first-remission ovarian cancer patients were enrolled from January 2011–Septermber 2013. Three dose levels were planned (25, 50, 100 mcg) with three cohorts of six patients each, with an additional 6-patient expansion cohort at the MTD. ELISA serologic IgM and IgG responses for each antigen was defined as positive response if antibody titers were ≥1:80 over the respective patient’s pre-vaccination serum. The study would be considered positive if at least four of 12 patients treated at the MTD showed immune responses for at least three of the five antigens. Twenty-four patients (median age, 54 years [range, 36–68]) were included in the safety analysis. Histology was high-grade serous in 22 patients (92%); 18 had stage III and six stage IV disease. The vaccine was well-tolerated at all doses, with no DLTs. At the highest treated dose, IgG and/or IgM responses were recorded against ≥3 antigens in 9/12 patients (75%), ≥4 in 7/12 (58%), and 5 in 3/12 (25%). With a median follow-up of 19 months (range, 2–39), 20 patients (83%) recurred and six (25%) died. The unimolecular pentavalent vaccine construct was shown to be safe and immunogenic. Such a construct greatly simplifies regulatory requirements and manufacturing, facilitates scalability, and provides adaptability.

## 1. Introduction

The majority of patients with advanced epithelial ovarian carcinoma will enter clinical remission following optimal primary surgical cytoreduction and completion of platinum- and taxane-based chemotherapy. Most however will relapse and ultimately develop chemoresistance [1,2]. Effective consolidation or maintenance strategies are needed to prevent recurrence or prolong remission. Immune-directed therapy represents an attractive investigational treatment strategy for patients who are in clinical remission [3]. Preclinical models have shown the clearance of circulating tumor cells and the eradication of systemic micrometastasis with both passively administered and vaccine-induced antibodies [4,5]. Ovarian cancers express a wide array of cell-surface antigens, including the carbohydrate epitopes GM2, Globo-H, Lewis y (Le^Y^), sialyl Tn (sTn), and Thompson Friedreich antigen (TF) [6,7]. These antigens are overexpressed on ovarian cancer cells and were selected for investigation in our vaccine trials as their expression on normal tissues is largely limited to secretory borders and other luminal sites that are by and large inaccessible to the immune system.

We have previously demonstrated in a series of phase I mixed monovalent vaccine studies the safe induction of antibody responses to these individual antigens in vaccines [8,9,10,11,12]. Although monovalent vaccination has resulted in immunologic responses, the heterogeneity of tumor cell surface antigen expression intimates that a multivalent approach to generate a broader immune response would be preferable. Our initial approach evaluated a multi-molecular polyvalent vaccine containing GM2, Globo-H, Le^Y^, Tn-Mucin 1 (MUC1), Tn cluster, sTn cluster, and TF cluster, each of which conjugated to a carrier protein, keyhole limpet hemocyanin (KLH) and mixed with adjuvant saponin, QS-21 [13]. We demonstrated the safe induction of antibody responses to 5 of the 7 vaccine antigens, with antibody titers comparable to those produced in our monovalent vaccines containing Tn, sTn, TF, MUC1, and Globo-H and slightly lower titers induced by GM2. Analagous to the monovalent vaccine trials Le^Y^ demonstrated low immunogenicity and was not included in our unimolecular vaccine. A phase II randomized trial utilizing that vaccine construct has completed accrual in a Gynecology Oncology Group trial (NCT00693342), and clinical results are pending.

The preparation of such a multivalent multi-molecular vaccine product from the combination of the individual antigens requires the manufacture and conjugation of multiple independent components. This is challenging from both a technical and intellectual property standpoint. Regulatory requirements necessitate validation of each individual component of the vaccine mixture (done over a period of sequential phase I trials), increased amounts of carrier proteins are required (a fixed amount for each individual component), and the synthesis of each monovalent-KLH construct involves a low-yielding final conjugation step.

A unimolecular pentavalent vaccine bearing the antigens Globo-H, GM2, sTn, TF and Tn was chemically synthesized, conjugated to KLH, and evaluated in a preclinical setting in the presence of the immunological adjuvant QS-21 [14]. The order of the antigens with respect to KLH was dictated by ease of synthesis with most chemically challenging ones such as Globo-H and GM2 connected last. Based on the reported serological analyses by enzyme-linked immunosorbent assays (ELISA) and fluorescence activated cell sorting (FACS), it was concluded that the immunological properties of each antigen of the unimolecular construct were preserved. This supports the concept of evaluating unimolecular multiantigenic synthetic vaccines in a phase I clinical trial. Simplifying the manufacturing and enhancing the scalability of the vaccine are a few advantages brought by uniting all antigens into one molecule. Conjugating one construct bearing multiple antigens to KLH adds an immense ease in regulatory terms. Additional antigens can be included in the vaccine peptide backbone thereby facilitating faster evaluation and manufacturing of new antigens as they are identified.

## 2. Experimental Section

### 2.1. Eligibility Criteria

Eligible patients had stage IV or “high-risk” stage III disease with histologically documented epithelial ovarian carcinoma arising in the ovary, fallopian tube, or peritoneum. Primary treatment must have included cytoreductive surgery and a platinum-based chemotherapy regimen, surgery, and suitability to enter a period of observation. “High risk” was defined by clear cell histology, suboptimal debulking, and/or failure to normalize CA-125 by the third cycle of chemotherapy. Complete clinical remission was defined as serum CA-125 less than or equal to 35 IU/mL, negative physical examination, and no objective evidence of disease on computed tomography (CT) of the abdomen and pelvis. Patients could have asymptomatic ≤1 cm soft tissue abnormalities on CT scan not considered definitive evidence of disease.

Other requirements included Karnofsky Performance Status ≥80; adequate organ function, defined as absolute neutrophil count ≥1000 cells/mm^3^, platelets ≥100,000 cells/mm^3^, and serum creatinine ≤1.5 times institutional upper limits of normal; and total bilirubin, aspartate transaminase (AST), and alkaline phosphatase ≤2.5 times institutional upper limits of normal. Given the concern for the possibility of autoimmune-related adverse effects, patients were also required to have thyroid-stimulating hormone (TSH) within institutional limits and a negative stool hemoccult (or negative endoscopic evaluation if positive). Patients were ineligible if they had a known autoimmune disease (excluding treated hypothyroidism) or allergy to shellfish.

### 2.2. Treatment Plan

The administered unimolecular pentavalent vaccine contained Globo-H, GM2, sTn TF, and Tn on a peptide backbone conjugated to KLH and mixed with adjuvant QS-21 (100 µg). Patients were scheduled to receive five vaccinations subcutaneously at weeks 1, 2, 3, 7, and 19. Six patients were treated in each cohort at one of three different dose levels (25, 50, and 100 mcg). No dose modifications were permitted. Prior to and during therapy, blood samples were drawn to assess safety and immune response of the vaccine.

### 2.3. Vaccine Preparation

**Figure 1:**
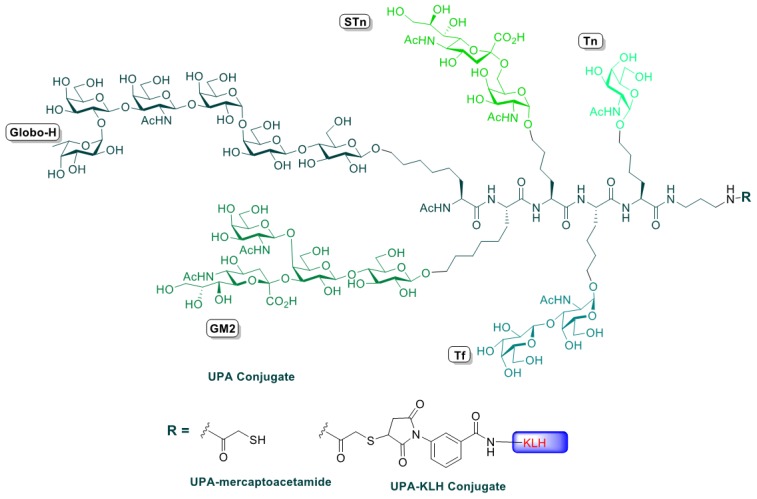
Unimolecular pentavalent vaccine.

The synthesis of the unimolecular pentavalent construct was performed through a well-established chemical assembly of what we have coined “cassettes”, referring to the carbohydrate epitopes Globo-H, GM2, sTn, TF and Tn, which were connected to properly functionalized amino acid side chains. From a chemical perspective, this synthetic assembly equates to a pentapeptide synthesis, with each amino acid side chain bearing an antigen, as previously described [15]. The fully glycosylated polypeptide backbone is shown in Figure 1.

Conjugation of the pentavalent antigen was conducted in the Clinical Grade Production Facility in house. KLH was obtained from the Sigma Chemical Company (Saint Louis, MO, USA). Covalent attachment to KLH was achieved in two stages by first reacting m-maleimidobenzoyl-*N*-hydroxysuccinimide ester (MBS) with free lysine side chains on the surface of KLH and then coupling the free thiol (SH) group of terminal mercaptoacetamide carrying the unimolecular penta-antigen to the maleimide and QS-21—a purified saponin fraction adjuvant obtained from Antigenics Corporation (now Agenus Inc., Lexington, MA, USA)—and mixed with the pentavalent antigen conjugate at the time of vaccine vialing. The dose of QS-21 was 100 mcg.

The final pentavalent antigen-KLH plus QS-21 was combined, sterile filtered, and vialed. The product was then tested for sterility, endotoxins, immunogenicity, and safety as stated in our Investigational New Drug (IND) application. Vials were stored at –80 °C by the Memorial Sloan Kettering Investigational Pharmacy until administration. 

### 2.4. Dose Adjustment and Toxicity Evaluation

Dose reduction or delay of vaccination was not permitted. Toxicity was evaluated according to the National Cancer Institute CTCAE scale version 4 [16]. Patients were removed from the study for a vaccine-related dose limiting toxicity (DLT) as defined by: ≥grade 2 allergic reaction (with the exception of fever), ≥grade 2 autoimmune reaction requiring treatment other than immunosuppressive drugs, ≥grade 3 autoimmune reaction, ≥grade 3 hematologic or non-hematologic toxicity including fever, or a ≥grade 3 injection site reaction. Any patient with grade 2 or greater toxicity was followed with appropriate studies until results returned to baseline. Patients were removed from study for disease progression as defined by RECIST 1.1.

### 2.5. Evaluation during Study

Pretreatment evaluation included a complete medical history, physical and radiologic examination (CT or MRI), vital signs, KPS assessment, and clinical laboratory tests, including hematologic, biochemistry, CA-125, and immunologic testing. Patients had repeat complete blood cell counts and a comprehensive biochemistry panel at regular intervals and at the off-study visit. Patients had vital signs recorded prior to and once after each vaccination. CT/MRI imaging was performed every three months while on study or sooner to evaluate patients if signs or symptoms, blood tests, or physical examination suggested disease progression. Serologic IgM and IgG antibody responses were measured by ELISA against each antigen at baseline, throughout the vaccination phase, and at the off-study follow-up.

### 2.6. Statistical Considerations

The endpoints of this pilot trial were safety and confirmation of immunogenicity of the vaccine. No systemic toxicity has occurred with the administration of similar vaccines at our center. Toxicity was not expected with this preparation relative to vaccination. The plan was to accrue six patients at each of the three dose levels (25 mcg, 50 mcg, and 100 mcg) followed by an expansion cohort of an additional six patients at the maximum tolerated dose (MTD). Toxicity was captured using CTCAE version 4.0. Escalation to the next higher dose level was based on no more than one DLT observed at a given dose level up to week 7 among six patients. If 0 or 1 DLT was observed, then escalation to the next dose level occurred. If two patients experienced a DLT, then the lower dose level was considered the MTD. The protocol stopped accrual when the MTD was determined and expanded appropriately.

### 2.7. Serological Analysis

We also sought to evaluate the immunogenicity of the vaccine. Serologic IgM and IgG antibody responses were measured by ELISA in duplicate against each antigen at baseline (pre-sera), sera obtained prior to each vaccination, and, if feasible, at the off-study visit as described [13]. Globo H-biotin, GM2-bioin, sTn-biotin, TF-biotin, or Tn-biotin was added separately to NeutrAvidin-coated ELISA plates (Pierce, Rockford, IL, USA). ELISA plates were incubated at 4°C overnight and blocked in 1% HSA in PBS buffer. Serially diluted patient’s sera was added to each well and incubated for 1 h at 37 °C and washed in PBS-0.05% Tween 20. Goat anti-human IgM or IgG labelled with alkaline phosphate secondary antibodies was added to the plates, incubated 20 min, washed, developed, and read at 405 nm on the ELISA plate reader (Microplate reader model 550 Bio-Rad, Hercules, CA, USA). The antibody titer was defined as the highest serum dilution showing an absorbance of ≥0.1 OD. In prior trials, antibodies were generally present by week 7 [13]. The same criteria for immunogenicity were used as those of the individual pilot trials: patients had to have IgM titer >1:80 or at least a four-fold increase in prevailing antibody titer if present at baseline. The proportion of responders was estimated for each dose level. If at least four of 12 patients treated at the highest dose level met these criteria for three or more antigens based on the immune response criteria, the study would be considered positive [17]. This calculation assumes that the probability of immune response under the null hypothesis (*i.e.*, no activity) is 0.1 *versus* the alternative hypothesis (*i.e.*, target response probability) is 0.5. Type I and Type II errors were set to 0.1.

While not an exploratory end-point of this study, progression-free survival (PFS) was recorded using the Kaplan-Meier method. PFS was defined as the time from the end of adjuvant chemotherapy until the time of disease progression as evaluated by RECIST criteria on imaging or by CA-125 criteria. Treatment failure by CA-125 was defined as CA-125 elevation to twice the upper limit of normal, confirmed by a second sample.

## 3. Results

### 3.1. Patient Characteristics

Twenty-four patients with advanced-stage, poor-risk ovarian carcinoma were enrolled on clinical trial NCT01248273 between 01/2011 and 09/2013. Patient characteristics are shown in Table 1. Patients had a median age of 54 years (range, 36–68 years). Primary sites included the ovary, 20; fallopian tube, three; and peritoneum, one. KPS ranged from 80–100%. The majority of patients were Caucasian (*n* = 22, 92%), and two patients were Asian (8%). Eighteen patients (75%) had stage III disease, and the remaining 6 (25%) had stage IV disease at diagnosis. Twenty-two patients (92%) had high-grade serous histology and two patients (8%) had clear cell histology.

### 3.2. Adverse Events

All 24 patients were included in the safety analysis. The vaccine was well tolerated, without any DLTs. Side effects were limited, and mild fatigue, fever, nausea, arthralgia, myalgia, rash and, localized injection site reactions were the most frequent. Table 2 depicts the maximum toxicity for treatment-related events with vaccine. No clinically relevant hematologic abnormalities were noted. No clinical or laboratory evidence of autoimmunity was seen.

### 3.3. Immune Response

**Figure 2:**
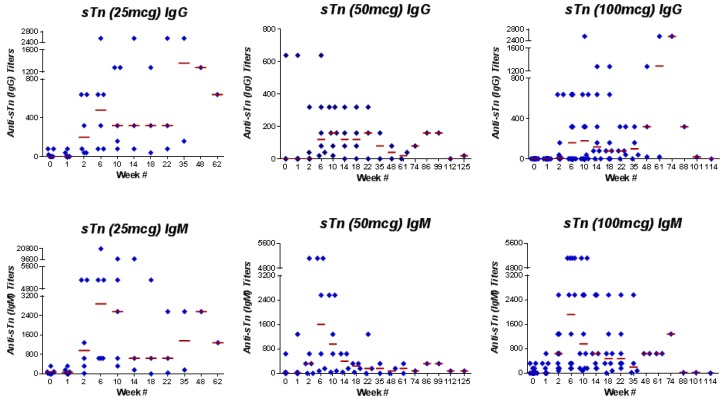
Anti-sTn (IgG and IgM) antibody titers of patients vaccinated with the unimolecular pentavalent-KLH vaccine.

IgG and/or IgM responses to at least three antigens in the vaccine were recorded in 20 (83%) of 24 patients treated on the study and in nine (75%) of 12 patients treated at the highest dose level, meeting the predetermined study endpoint. Seven (58%) of the 12 patients treated at the highest dose level developed responses to at least four antigens, and three patients (25%) responded to all five antigens contained in the vaccine. One patient did not meet the criteria for immune response against the antigens. Individual IgM and/or IgG responses recorded in patients treated at the highest dose level were as follows: Globo-H, 7 (58%); GM2, 4 (33%); sTn, 11 (92%); TF, 9 (75%); and Tn, 10 (83%). The IgM and IgG responses to these antigens are depicted in Table 3. Each antigen met our previously determined definition of “positive”, with GM2 being the least immunogenic and sTn associated with the greatest number of responders. IgG and IgM responses for sTn are illustrated in Figure 2.

### 3.4. Progression-Free Survival

**Figure 3:**
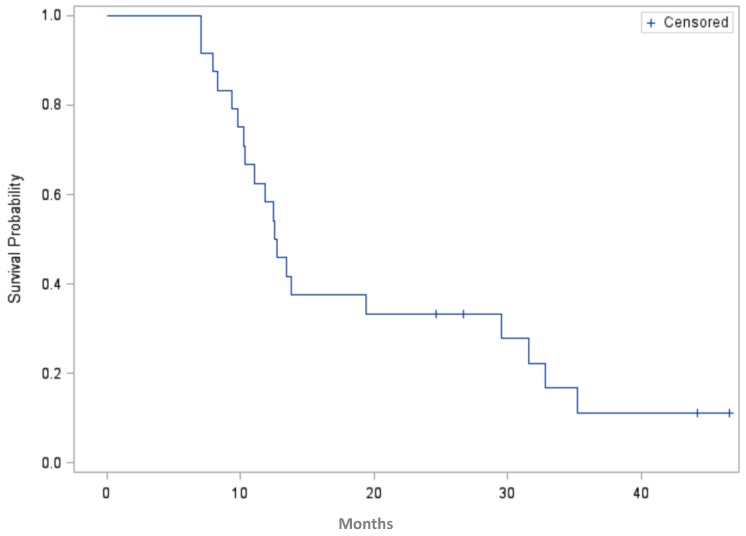
Progression-free survival in months for patients enrolled on the study (*n* = 24, four patients are censored).

Although clinical outcome was not the study end point of this phase I study, the PFS is illustrated in Figure 3. PFS was calculated for 20 events and censored at 24.6, 27, 42 and 46.5 months for four patients who continued in remission at last follow-up. The small patient numbers preclude any correlation of antibody response generated with PFS. It was observed that one of the patients with the longest PFS generated a response to all five antigens. At last follow-up, six patients had died of disease and the remaining 18 were alive. For this high-risk group, the median PFS was 12.6 months (95% CI, 10.2–29.5 months) from start of adjuvant chemotherapy. 

## 4. Discussion

Our results show that it is feasible to safely induce antibody responses against five ovarian cancer cell surface antigens—Globo-H, GM2, sTn, TF, and Tn—using a unimolecular pentavalent-KLH antigen construct with QS-21 as the immune adjuvant. The five individual components had been tested separately in previous trials and their safety confirmed; however, there was concern that the unimolecular pentavalent vaccine construct might be associated with increased toxicity or less immunogenicity. In our study, we show that all but one of the 24 (96%) vaccinated patients had antibody responses against at least one of these antigens, and 20 patients (83%) responded against at least three antigens after vaccination. IgM antibody titers against the three antigens sTn, TF, and Tn met the predetermined study endpoint after vaccination with the pentavalent vaccine (sTn, 1:1,280; TF, 1:1,280; and Tn, 1:2,560), as did those generated in a previously reported trial of patients treated with the monovalent-KLH vaccine conjugate plus QS-21 (sTn, 1:80; TF, 1:640; and Tn, 1:160) [13]. Clinical outcome was not the endpoint of this phase I trial. The PFS in this study is within the range of reported values for this high-risk population (clear cell, stage 4, suboptimal, failure to normalize CA125). The patient with the longest PFS generated an IgM response to all five antigens in the vaccine and the single patient who did not mount a response to any of the antigens had the shortest PFS. While these observations may serve as hypothesis-generating, the small sample size obfuscates any comparisons of time to treatment failure to the differences in immune titers. In point of fact, five of the six study patients who have died had each produced responses to four of the vaccine antigens. Although we have shown that the construct is immunogenic with regards to antibody thresholds, it is possible that there are quantitative differences in the antibody response between the unimolecular approach and our previous studies. This could be related to the position of the antigen in relation to KLH based on steric hinderance, and a consideration of repositioning antigens in any future studies would be reasonable.

No DLTs were seen. Toxicity was restricted to local erythema, pain, and induration at vaccination sites in 21 patients (88%), as well as flu-like symptoms in the occasional patient, all known to be consequences of the 100-mcg dose of QS-21. Mild grade 1 elevation in transaminases was noted, but no clinically significant alterations in liver function, occult blood in the stool, or other abnormalities were detected.

## 5. Conclusions

The unimolecular vaccine was shown to be safe and immunogenic. Nine (75%) of 12 patients treated at the highest dose of 100 mcg and 20 (83%) of all 24 patients treated on the study responded to at least three antigens. This immune response was comparable to our previously reported immune response in a phase I trial of a heptavalent vaccine with individual antigens conjugated to KLH [13]. This unimolecular construct greatly simplifies manufacturing, permits the addition or exchange of multiple new antigens, and allows for easy scalability.

## Figures and Tables

**Table 1:** Baseline patient demographics (*N* = 24).

Variable	Number of Patients (%)
Median age (Range)	54 years (36–68)
FIGO * Stage at diagnosis	
III	18 (75%)
IV	6 (25%)
Cancer type	
Ovarian	20 (83%)
Primary Peritoneal	1 (4%)
Fallopian Tube	3 (13%)
Median Karnofsky Performance Status (range)	90 (80–100)
Race	
Caucasian	22 (92%)
Asian	2 (8%)
Histologic subtype	
High-grade serous carcinoma	22 (92%)
Clear cell	2 (8%)

* FIGO = International Federation of Gynecology and Obstetrics.

**Table 2:** Patients per maximum toxicity grade for ≥ grade 2 treatment-related events (*n* = 24).

Treatment-Related Adverse Events	Grade 2 n (%)	Grade 3 n (%)	Grade 4 n (%)
Injection site reaction	2 (8%)	0	0
Hyperglycemia	2 (8%)	0	0
Hypoglycemia	1 (4%)	0	0
Thrombocytopenia	1 (4%)	0	0
Neutropenia	1 (4%)	0	0
Leukopenia	1 (4%)	0	0
Elevated alanine aminotransferase	1 (4%)	0	0
Fever	1 (4%)	0	0

**Table 3:** Serologic IgM and IgG responses to antigens at the highest dose level tested (*n* = 12).

Antigen	IgM Response n (%)	IgG Response n (%)
GM2	3 (25%)	2 (17%)
Globo-H	1 (8%)	7 (58%)
Tn	7 (58%)	10 (83%)
TF	8 (67%)	3 (25%)
sTn	11 (92%)	8 (67%)
